# Cohort retrospective study: the neutrophil to lymphocyte ratio as an independent predictor of outcomes at the presentation of the multi-trauma patient

**DOI:** 10.1186/s12245-020-0266-3

**Published:** 2020-02-04

**Authors:** Soulaiman Elias Soulaiman, Dalal Dopa, Al-Batool T. Raad, Walaa Hasan, Niyazi Ibrahim, Al-Ykzan Hasan, Hussam Aldin Sulaiman, Moufid Darwich

**Affiliations:** 1 Department of Hematology and Bone Marrow Transplantation, Tishreen Hospital, Damascus, Syria; 2 Department of Laboratory, Tishreen Hospital, Damascus, Syria; 3 Department of Surgery, Tishreen Hospital, Damascus, Syria; 4 Department of Orthopedic Surgery, Tishreen Hospital, Damascus, Syria

**Keywords:** Neutrophil to lymphocyte ratio, Trauma, Intensive care, Mortality

## Abstract

**Background:**

Although the association of neutrophil to lymphocyte ratio (NLR) with mortality in trauma patients has recently been shown, there is a paucity of research on the association with other outcomes. Recent studies suggest that the NLR has a predictive value of mortality in trauma patients during various times of admission. This study aimed to determine the prognostic impact of NLR at the presentation in critically ill trauma patients.

**Methods:**

A retrospective cohort study of adult trauma patients between July 2017 and November 2017 in Tishreen Hospital. All patients who had arrived at the emergency department with multi-trauma injury within the age category (14–80 years) were included in this analysis. The prophetical capability of NLR on mortality was assessed by the receiver operative characteristics (ROC) curve. To identify the impact of the NLR on survival, a separate log-rank test was used. Multivariable Cox proportional hazard modeling was used to identify independent predictors of mortality.

**Results:**

Throughout the time of the study, 566 patients met the inclusion criteria. Of these, 98.8% were male, 75.8% sustained penetrating trauma, and median age [IQR25–IQR75] was 26 [23–32]. Ninety-seven patients (17.1%) had major trauma, with an Injury Severity Score (ISS) ≥ 15. Using the ROC curve analyses hospitalization day 1, optimal NLR cutoff values of 4.00 were calculated by maximizing the Youden index. Kaplan-Meier curves revealed an NLR greater than or equal to these cutoff values as a marker for increased in-hospital mortality (*p* = 0.020, log-rank test). The Cox regression model demonstrated significant collinearity among the predictive variables (all VIF results < 2). Only ISS > 15 has a significant statistical relation with elevated NLR on day 1 (*p* = 0.010).

**Conclusions:**

Elevated NLR on day 1 has high predictive power for overall survival during the first 30 days after trauma, but it was not independent of other factors.

## Background

### Injuries

According to the World Health Organization, injuries are still a major cause of severe disability and death. Trauma-related injuries result in the death of more than five million people worldwide annually accounting for approximately 9% of global mortality [1]. The care of severely injured patients remains a challenge, which makes trauma death studies have importance as these proceed with a medical audit and a measure for quality of care provided to trauma patients within the prehospital and in-hospital setting.

The classical trimodal distribution of trauma deaths describes three peaks of deaths following trauma: immediate, early, and late deaths [2]. The predominant cause of death after trauma continues to be central nervous system (CNS) injury, followed by exsanguination, while sepsis and multi-organ failure (MOF) continue to be predominant causes of late death [3]**.**

### Prognosis and risk factors

One of the important factors that increase mortality in post-traumatic patients is pathological pro- and anti-inflammatory responses occurring in the first hours after extensive trauma with massive infection, which is still difficult to control and to discriminate from a physiological immune response [4]. In recent years, there has been a particular focus on the balance of this pro-inflammatory response and the anti-inflammatory response as systemic predictors of outcomes in critically ill patients. Patients often develop numerous abnormalities in their host defense systems in response to severe injury [5]. Problems arise when this unbalanced inflammatory response escalates and releases an excess of pro-inflammatory mediators such as IL-1, IL-6, IL-8, and TNFα, culminating in systemic inflammatory response syndrome (SIRS) [6]. Progression of this uncontrolled cytokine cascade and hyper-inflammation augments the injury burden, resulting in deleterious and often life-threatening events such as sepsis and multiple organ dysfunction syndrome (MODS).

### The neutrophil to lymphocyte ratio

In contrast to the widespread activation of neutrophils, post-injury is the fall in total lymphocyte levels which occurs in response to multiple trauma. The prognostic value of neutrophil to lymphocyte ratio (NLR) had estimated in non-traumatic disorders where studies showed NLR is effective in the diagnosis of familial Mediterranean fever [7]; it has also been studied for acute appendicitis and has been found to have a higher sensitivity than leukocytosis [8]. For this reason, the NLR can potentially be used as an early indicator of inflammatory homeostasis derailment in patients with tissue injury.

### Aim of the study

We conducted this study in trying to assess the NLR in multi-trauma patients at presentation as a useful predictor of worse outcomes, and then it can be used to help build a prognostic model.

## Methods

### Study design

This study is a retrospective cohort study of adult trauma patients between July 2017 and November 2017 in Tishreen Hospital. All patients who had arrived at the emergency department with multi-trauma injury within the age category (14–80 years) were included in this analysis.

All patients coming directly from the site of injury or referred from another hospital to the emergency department were subjected to the same criteria after excluding those who spent more than 24 h to arrive in our hospital. During war conditions, it was difficult to obtain accurate data about the initial approach of the injured due to the enormous workload and the difficulty of mobility where the priority was to save lives.

Patients were excluded if they were below 14 years of age because there is a wide variation in the number of lymphocytes within this age group that may affect the estimation for the prognostic value of NLR. We also excluded patients who had expired in the emergency department or the operating room because it was difficult to get all variables that we need for our study given the change in the value of NLR during the hospitalization period and its relationship with other variables.

For the purpose of the study, two trauma team experts, one collected laboratory data and other followed clinical and surgical issues, performed extensive chart and computer checks for data completeness, accuracy, and consistency.

Demographic and clinical data elements from the respective medical files collected by trained chart abstractor then computerized into database excel file included age, gender, Injury Severity Score (ISS), mechanism of injury (MOI), initial vital signs upon presentation to the emergency department (ED), hospital length of stay (LOS), intensive care unit LOS (ICU LOS), blood cell count with differential, and in-hospital mortality.

#### Primary outcome variable

The primary variable is the NLR at admission (day 1), which is defined as the ratio of absolute neutrophil to absolute lymphocytes taken from the data of the laboratory department at the admission in the emergency department.

We considered cutoff for NLR equal to 4 to study patients in two groups to determine the prognostic value of this variable on the overall survival and mortality.

#### Secondary outcome variables

The secondary variables are age; gender; ISS, calculated by a scientific calculator by entering the required data after obtaining it from the patient files; MOI, with two types of injuries identified as penetrating and blunt; initial vital signs upon presentation to the ED; LOS; and ICU LOS, aiming to build epidemiological study and screen for the risk factors.

#### Statistical analysis

The data were analyzed by the IBM SPSS v.22 program for the normal distribution of continuous variables by using histograms and the Shapiro-Wilk test. Continuous variables were reported as medians and interquartile ranges (IQR). Categorical variables were reported as frequencies and percentages. To examine the differences between NLR groups, categorical covariates were analyzed using Fisher’s exact test, while the continuous covariates were analyzed based on Mann-Whitney *U* test. The predictive capacity of the NLR on mortality was assessed using receiver operating characteristics (ROC) curve analysis. The NLR analysis was performed for hospital day 1. These periods were selected to adjust for the clinical probability of early and late complications. Optimum cutoff values were determined on the ROC curve with the maximum Youden index [sensitivity-(1-specificity)]. Baseline characteristics and outcome variables of the high and the low NLR groups were compared in a univariate analysis. The in-hospital mortality during the study period was assessed using Kaplan-Meier curves, and the log-rank test was used for between-group comparisons for the NLR cutoff values on hospital day 1.

## Results

During the study time frame, baseline characteristics of patients are shown in Table 1: a total of 566 trauma patients aged 14 and older were admitted to an emergency department in Tishreen Hospital, and patients arrived in the hospital by two methods: directly from the place of accident or transferring from another hospital. Five hundred and sixty (98.8%) were male. Median age [IQR25–IQR75] was 26 [23–32], and 42.2% of patients were older than 26 years. Penetrating injury was seen in 429 (75.8%) of the patients, and 97 (17.1%) patients had major trauma, with an ISS ≥ 15. Only six patients (1.1%) had damage control surgery (DCS).

**Table 1:** Baseline characteristics of patients admitted to Tishreen Hospital during the study period

	*N* = 566
Age > 26	239 (42.2%)
Male gender	506 (98.8%)
ISS > 15	97 (17.1%)
Penetrate injury	429 (75.8%)
LOS > 7 days	73 (12.9%)
ICU admission	65 (11.5%)
ICU LOS > 7 days	23 (4.1%)
SBP < 90	97 (17.1%)
DCS	6 (1.1%)

*ISS* Injury Severity Score, *LOS* length of stay in hospital, *ICU* intensive care unit, *SBP* systolic blood pressure, *DCS* damage control surgery

All patients (566) had hospitalization, 12.9% of them (*n* = 73) were hospitalized for more than 7 days, 65 patients needed ICU, and 4.1% (*n* = 23) of these patients stayed in ICU for more than 7 days. At admission to the emergency department, only 17.1% (*n* = 97) had systolic blood pressure less or more than 90 mm.

**Fig. 1:**
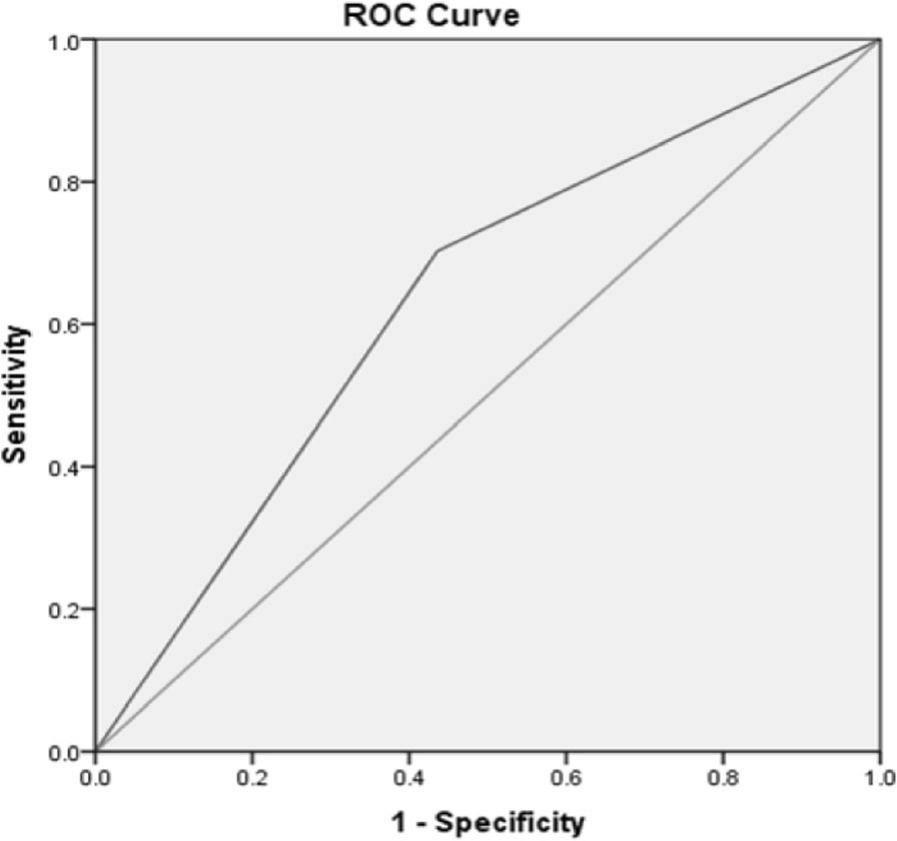
The ROC curve analysis of neutrophil to lymphocyte ratio (NLR) at time admission (day 1) revealed optimal cutoff values of 4, corresponding to a sensitivity of 70.3% and a specificity of 56.4%

For the NLR on day 1, the AUC was 0.633 (95% CI 0.542–0.725, *p* = 0.080). The ROC curve analysis for hospital day 1 revealed optimal cutoff values of 4, corresponding to a sensitivity of 70.3% and specificity of 56.4% (Fig. 1).

**Fig. 2:**
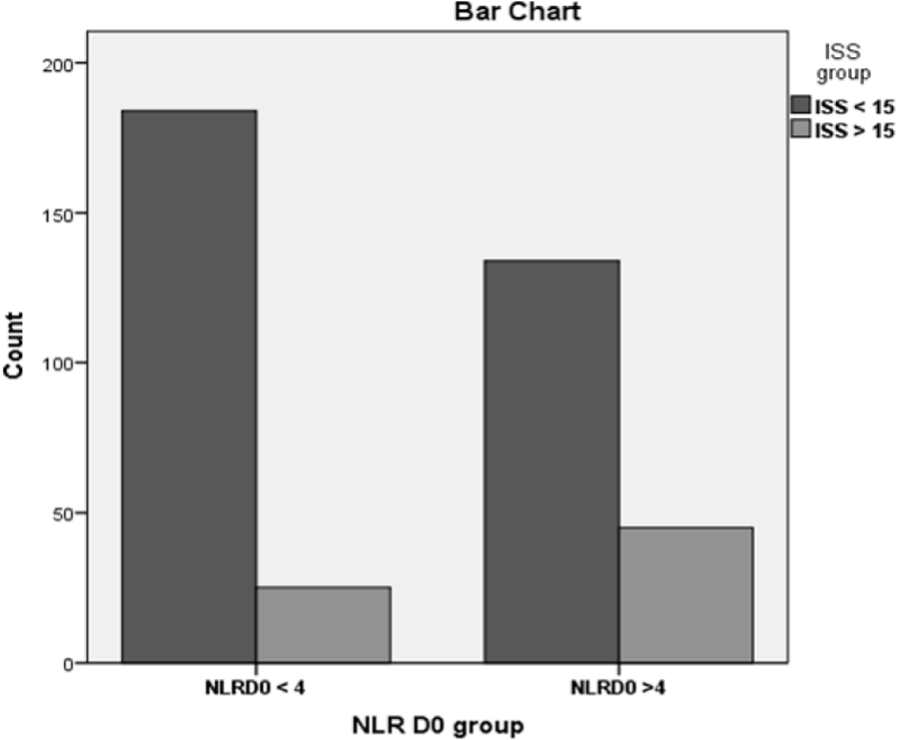
The relation between Injury Severity Score (ISS) and neutrophil to lymphocyte ratio (NLR) at admission (day 1); ISS > 15 has statistical significance (*p* = 0.010)

When baseline characteristics were compared (Table 2), for the high and low NLR groups on hospital day 1, in the high NLR group, age ≥ 26 years (*p* = 0.758), systolic blood pressure (SBP) ≤ 90 mmHg (*p* = 0.662), penetrating injury (*p* = 1.000), lengthy stay in hospital LOS > 7 days (*p* = 0.228), length stay in ICU > 7 days (*p* = 0.229), all have no statistical significance between high and low NLR groups. Only ISS > 15 (*p* = 0.010) has statistical significance (Fig. 2).

**Table 2:** The relation between baseline characteristics and neutrophil to lymphocyte ratio (NLR) at admission (day 1)

	NLR > 4 on day 1 (*n*, 165)	NLR < 4 on day 1 (*n*, 184)	*p* value
Age > 26 years	74 (41.3%)	105 (58.7%)	0.758
ISS > 15	45 (25.1%)	25 (12.0%)	0.010*
Penetrate injury	134 (74.9%)	156 (74.6%)	1.000
LOS > 7 days	28 (15.6%)	23 (11%)	0.228
ICU LOS > 7 days	12 (7.5%)	5 (2.7%)	0.229
SBP < 90	166 (94.9%)	201 (96.2%)	0.662

*ISS* Injury Severity Score, *LOS* length of stay in hospital, *ICU* intensive care unit, *SBP* systolic blood pressure

*statistical significance

**Fig. 3:**
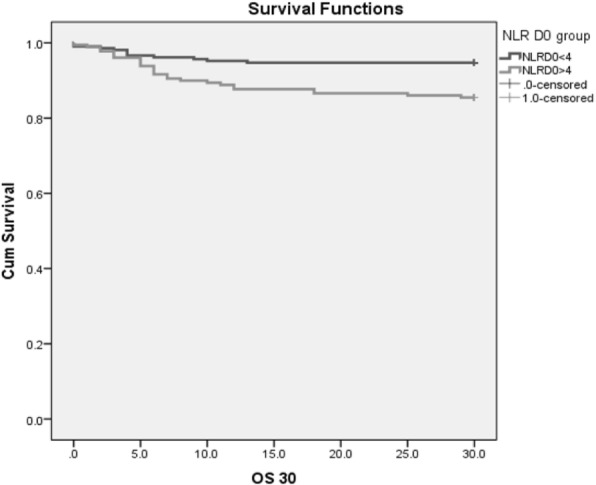
Kaplan-Meier curves of overall survival (OS) according to neutrophil to lymphocyte ratio (NLR) at admission (day 1); group with NLR > 4 had OS 85.5% and group with NLR < 4 had OS 94.7% during the first 30 days of trauma. *p* value 0.020

When the Kaplan-Meier curves (Fig. 3) of the overall survival were compared, a statistically significant difference was observed between the patients above and below the NLR cutoff values for day 1 hospitalization (*p* = 0.020).

The regression models demonstrated significant collinearity among the predictive variables (all VIF results < 2). Significant interactions were identified between the NLR, time, and the other predictive variables (including age ≥ 26, SBP ≤ 90, MOI penetrate vs blunt, ISS > 15).

## Discussion

Outcome prediction in the critically ill trauma patients using readily available, easily obtainable, simple, and repeatable clinical data points is an important research goal that to date has not been adequately achieved. NLR is a simple, stand-alone method for evaluating systemic inflammation and outcomes in trauma patients.

During war conditions, the number of injuries coming to the emergency department is increasing, and the types of injury vary beyond the ability of the medical staff to deal decisively and effectively with all cases. In such a situation, mostly, the first step is categorizing and managing the injuries according to their risk. To achieve this step, we need an easy, fast, and credible prognostic scale. A review of the literature found several studies on prognostic factors for trauma patients, which suggested that the predictive capacity of the well-known scoring systems such as the admission ISS are not as good as the several well-validated physiologic-based scoring models such as the Denver, Sequential Organ Failure Assessment (SOFA), Acute Physiology and Chronic Health Evaluation II (APACHE II), Revised Trauma Score, and Trauma and Injury Severity Score (TRISS) [9]. However, the predictive capacities of these physiologic-based models are complicated and too unwieldy for daily use and require a long time to apply.

Few studies have evaluated the association between NLR and outcomes in multi-trauma patients, demonstrating worse outcomes with increasing NLR. A recent study had found that a trajectory of increasing NLR over the first 48 h of admission is associated with the development of organ failure among male trauma patients [10]. Another study realizes that NLR is strongly associated with early mortality in trauma patients with severe hemorrhage managed with multi-transfusion protocol (MTP) [11]. According to Dilektasli et al. who demonstrated the prognostic role of NLR at days 2 and 5 in predicting hospital deaths in trauma patients, in contrast, the NLR in the first 24 h was not useful for predicting outcomes when compared with the next days in the surgical intensive care unit [12]; in this study, hospital days 2 and 5 revealed optimal cutoff values of 8.19 and 7.92, corresponding to a sensitivity and specificity of 70.8% and 61.9% and 75.6% and 66.9%, respectively. According to Forget et al., BMC Res Notes, the normal NLR values in an adult, non-geriatric, population in good health are between 0.78 and 3.53 [13]. So, our study for hospital day 1 revealed optimal cutoff values of 4, corresponding to a sensitivity of 70.3% and specificity of 56.4%.

Our study showed an important prognostic value for NLR during the first 24 h of receiving patients in the emergency department to predict survival within 30 days following the admission.

## Conclusion

Elevated NLR during the first 24 h of admission (day 1) has high predictive power for overall survival during the first 30 days after trauma, but it was not independent of other factors.

We found a closed relation between a high level of ISS and elevated NLR on day 1 which guided for more studies in trying to build a new prognostic model for multi-trauma patients which included ISS and NLR at admission. To achieve that, a prospective external validation is warranted in a large trauma population.

This study is a retrospective database study; the nature of this study may lead to error due to confounding and bias for patient and treatment selections.

## Data Availability

The datasets used during the current study are available from the corresponding author on a reasonable request.
